# Recent advances in biomedical applications of accelerator mass spectrometry

**DOI:** 10.1186/1423-0127-16-54

**Published:** 2009-06-17

**Authors:** Sang Soo Hah

**Affiliations:** 1 Department of Chemistry and Research Institute for Basic Sciences, Kyung Hee University 1 Hoegi-dong, Dongdaemun-gu, Seoul 130-701, Korea

## Abstract

The use of radioisotopes has a long history in biomedical science, and the technique of accelerator mass spectrometry (AMS), an extremely sensitive nuclear physics technique for detection of very low-abundant, stable and long-lived isotopes, has now revolutionized high-sensitivity isotope detection in biomedical research, because it allows the direct determination of the amount of isotope in a sample rather than measuring its decay, and thus the quantitative analysis of the fate of the radiolabeled probes under the given conditions. Since AMS was first used in the early 90's for the analysis of biological samples containing enriched ^14^C for toxicology and cancer research, the biomedical applications of AMS to date range from *in vitro *to *in vivo *studies, including the studies of 1) toxicant and drug metabolism, 2) neuroscience, 3) pharmacokinetics, and 4) nutrition and metabolism of endogenous molecules such as vitamins. In addition, a new drug development concept that relies on the ultrasensitivity of AMS, known as human microdosing, is being used to obtain early human metabolism information of candidate drugs. These various aspects of AMS are reviewed and a perspective on future applications of AMS to biomedical research is provided.

## Introduction

**Figure 1:**
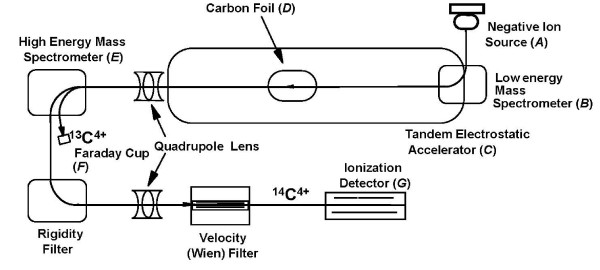
**Schematic diagram of an accelerator mass spectrometry (AMS)**. Cesium (Cs) sputter ion source (*A*) contains the wheel with the graphite samples under high vacuum. Atomic Cs vapor is produced from a heated Cs reservoir and sprayed on to a heated ionizer surface, producing Cs^+ ^ions that are accelerated towards the target held at -8 kV. The Cs^+ ^ions sputter carbon atoms and ions from the target that are ionized to C^- ^ions as they pass through a condensed Cs layer on the cathode. Negative ions at *m/z *13 (^13^C^-^) and 14 (^14^C^-^) are pulsed through an injection magnet or low energy mass spectrometer (*B*) into a tandem electrostatic accelerator (*C*). Negative C^- ^ions are accelerated towards the high-voltage terminal (+518 kV) in the center of the accelerator where they are converted to positive ions, C^+ ^being the most abundant. The high-energy ion beam is focused to collide with argon gas electron stripper or a thin carbon foil, 0.02–0.05 μm thick (*D*) in a collision cell. Molecular charged ions such as ^13^CH^- ^and ^12^CH_2_^- ^do not survive the electron stripping process and are converted to atomic species, and ^14^N^- ^ions decay on a femtosecond time-scale. The positive ions are repelled toward the high-energy exit of the accelerator held at 0 V. ^13^C^+ ^and ^14^C^+ ^ions are separated by momentum using a high-energy analyzing magnet or mass spectrometer (*E*). The beam currents of relatively abundant ^12^C and ^13^C are measured with Faraday cups (*F*). The ^14^C beam is focused by a quadruple and electrostatic cylinder analyzer and the atoms are counted in a gas ionization detector (*G*). The advantage of a gas ionization detector is that it measures energy loss in terms of Δ*E*/*E *which facilitates isotope separation. It is possible to optimize the detector to the energy-loss separation of the isotope.

Accelerator mass spectrometry (AMS; see Figure 1 for AMS schematic diagram) is an extremely sensitive nuclear physics technique for detection of very low-abundant, stable and long-lived isotopes, initially developed in the mid-70's as a method of determining isotope ratios for geochronology and archaeological research [1,2]. The technique utilizes a tandem van de Graaff accelerator in order to generate the potential energy, allowing for separation of elemental isotopes at the single atom level. Therefore, AMS can be applied to quantitating the concentrations of long-lived radioisotopes, such as ^14^C, for which decay counting is an inefficient method of quantitation because of its relatively long half-life of 5760 years [3]. Much of this discussion concerns ^14^C because it is the predominant isotope for biomedical/bioanalytical probe analysis. However, equivalent discussions refer equally well with changes in chemistry [4], to several other long-lived isotopes that can be quantitated by AMS: ^3^H, ^10^Be, ^16^Al, ^36^Cl, ^41^Ca, ^56^Ni, ^99^Tc, ^129^I, and ^239^Pu.

The sensitivity of AMS for radiocarbon can be emphasized by its use in geochronology or carbon dating of historical artefacts [1,2]. As widely known, small amounts of ^14^C are constantly being formed from ^14^N by bombardment with cosmic radiation in the upper atmosphere, and this formation gives rise to ^14^CO_2_, and maintains the atmosphere at a nearly constant radiocarbon concentration of about 1.2 × 10^-10^% ^14^C (primarily as CO_2_) [5-7]. Plants fix atmospheric ^14^CO_2_, animals eat the plants and thus all higher living organisms contain ^14^C in equilibrium. When an organism dies, there is no longer any carbon exchange, and ^14^C decays over time. Thus, the ^12^C:^14^C ratio can be correlated with the amount of time elapsed after an organism's death, which is the basis of carbon dating. Carbon dating now extends beyond 50,000 years back in time [3].

The most conventional method for the measurement of radioactivity for biomedical applications is liquid scintillation counting (LSC), a process known as decay counting. LSC, however, suffers from an innate insensitivity. In fact, it takes over a billion atoms of ^14^C to generate an average of only one disintegration per minute (dpm).

AMS, on the other hand, allows direct measurement of ^12^C and ^14^C atoms by physically separating them in an ion beam [3]. Since the atoms are measured directly, without a necessity to wait for a disintegration event to occur, AMS is about six orders of magnitude more sensitive than LSC. Nowadays, AMS is the method of choice for carbon dating as well as other LCS-utilized biomedical research because of the technique's sensitivity and precision. The workings of an AMS instrument are outlined elsewhere [8-10].

### The development of biomedical AMS

The use of radioisotopes has a long history in biomedical science. Isotopic enrichment of xenobiotics with ^14^C is routinely used as a method of following their metabolic fate in both animals and humans, and a drug is typically synthesized such that the natural abundance of ^14^C is increased from the background level of 1.2 × 10^-10^% to 20% or even higher depending upon the compound. The low energy β-radioactivity is then used to track the radiolabeled compound and its metabolites in biological samples derived from laboratory animal or human studies. LSC has been generally used for a long time to detect, follow and quantitate levels of radiotracer in such studies. There are occasions, however, when the low sensitivity of LSC becomes experimentally limiting, while the technique of AMS has now changed the experimental paradigm because its extremely sensitive detection limit virtually removed the previous experimental barriers.

The high sensitivity of AMS indeed affects experimental designs in several ways. First, the radioisotopic dose can be reduced to inconsequential levels of radiolysis, hazardous waste streams, and human subject exposure. Secondly, the chemical dose to a biological system, including humans of all ages and health status, is minimized to sub-physiological and sub-toxic doses. This allows a realistic analysis of the effects arising from low chemical doses. For example, children and women of child-bearing ages, who are important targets of increased health-related research, are suitable subjects at the low doses afforded by AMS [10,11], since the administration of such low levels of ^14^C are considered non-radioactive from a regulatory point of view. Finally, even if the sampled material needs fractionation to specific biomolecules prior to quantitation, the sample sizes are reduced to amounts that can be obtained from well-defined, and often non-invasive procedures.

For a practical AMS measurement, biological samples containing 0.2–5 mg of carbon must be converted to solid carbon (graphite or fullerene) using a two-step process [12]. In a quartz tube, and using excess copper oxide (CuO), the sample's biological carbon is oxidized to CO_2_. The CO_2 _is then reduced to solid carbon by both reduction with titanium hydride and zinc powder and catalyzation with either iron or cobalt. Because this process is independent of the chemical nature of the sample, it eliminates interference or suppression from other sample components. Therefore, AMS provides one piece of information about the sample of carbon measured: the precise ^12^C:^14^C ratio. In AMS, one measures the isotope ratio with respect to that of a well-known (external) standard in order to produce an absolute isotope concentration for the combusted sample [13,14]. With AMS, experimenters only need the fractional elemental abundance of the sample and the specific activity of the tracer compound in order to obtain, in the units most useful for interpretation, the concentration of the tracer in the sampled material. The mechanics of an AMS instrument, the mathematical conversions of the measured values to meaningful "Modern" values, and the comparisons with LSC are well reviewed in the literature [3,11,15-17].

For the first time in 1990, sensitive and precise quantitation of ^14^C was applied to the analysis of biological samples containing enriched ^14^C-labeled carcinogens for toxicology and cancer studies by Turteltaub *et al*. [18]. Their research quantified chemical binding of the ^14^C-labeled carcinogens to DNA at the level of 1 binding in 10^11 ^bases. The benefits of using AMS for the analysis of samples derived from radiotracer studies with humans soon became apparent, since AMS produces very specific quantitation with simple analysis [19]. Any isotope concentration greater than the known stable natural ^14^C background must arise from an introduced isotope label ("introduced" includes contamination, which must be carefully controlled and avoided). In the simplest experimental design, there is only one external radioactive source, perhaps a radiolabeled compound introduced into the biological system at a specific time. The isotope ratio of the isolated sample is then easily converted to the concentration of the labeled compound and its metabolites per g or ml of the analyte.

Not surprisingly, AMS has soon become a tool of choice for pharmacokinetic analyses [10,11,16]. All the metabolites of the compound that contain the labeled moiety can be directly quantified in chromatographic separations without resorting either to secondary standards or to prior knowledge of metabolic pathways. Although some fluorescent methods quantitate into the amol levels [20,21], they require derivatization procedures that are not suitable for *in vivo *tracing, create tracers that are not chemically equivalent, and are less general in applicability across many biological systems. Conversely, AMS is specific only to the labeled compound in any chemical or biological medium. Such specificity requires neither prior speciation nor the introduction of either molecular modifications or internal standards. With AMS, it is possible to conduct radiotracer studies in human with the administration of such low levels of ^14^C [10,11].

The most recent innovation using AMS technology is the so-called "microdosing" concept [10]. Choosing a drug for clinical trials from numerous candidates is very much a hit-and-miss business. Data are gathered from *in vitro*, *in vivo*, and *in silico *models in order to predict the drug's behavior in humans but such methods are probably only about 60% predictive. Presented with a choice of good candidates, it would be better to take them all into human subjects. This would, however, be prohibitively expensive, as each compound would require a significant package of toxicological safety testing. Alternatively, each candidate drug could be given to human volunteers at very low levels of a few tens, or at most a hundred μg. At these levels, only a limited toxicology package is required and *in vivo *human data can be acquired for candidate selection [22]. Only AMS has the required sensitivity to conduct such studies at the low μg level.

**Figure 2:**
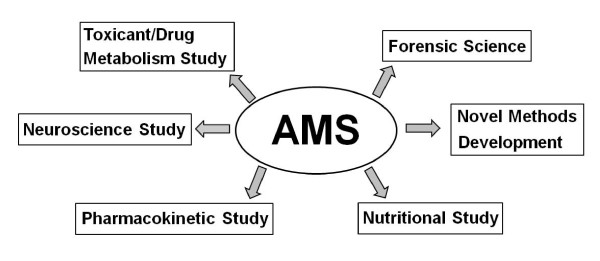
**Major applications of AMS in various biomedical investigations discussed in the review**.

In this review, the recent development of AMS methods to the present day in biomedical/bioanalytical research where it is being strategically used with high precision (see Figure 2 for the major applications of AMS discussed here) will be followed.

### Toxicant/drug metabolism study using AMS

After the first biomedical application of sensitive and precise quantitation of ^14^C by AMS in 1990 [18], the technique has been explored for using animals and for fractionating tissues, cells and molecules in the study of metabolism, covalent macromolecule-ligand interactions, and non-covalent macromolecule-ligand interactions with amol sensitivity [8-10]. For example, Phillips *et al*. reported in 2000 a good application of AMS for these purposes by performing metabolism and macromolecular binding studies, primarily of environmental toxicants and toxins, as well as of vitamins in humans at physiological concentrations [23], which combined two general areas into an ongoing study of cancer chemoprevention by dietary agents. They investigated covalent interactions of metabolic products with DNA and proteins, both in animal hosts and in humans.

The heterocyclic amines are compounds that are found in cooked meat and are potent carcinogens in rodent models. However, their role in human cancer remains largely unknown. Using AMS, Felton's group at the Lawrence Livermore National Laboratory has identified the metabolites of [2-^14^C]2-amino-1-methyl-6-phenylimidazo [4,5-b]pyridine (PhIP) in humans and the relationships between the activities of key enzymes involved in PhIP metabolism and metabolite profiles [24,25]. In addition, the levels of PhIP adducts on the DNA and blood proteins of humans and rodents were quantified by AMS [26,27]. Through these works, it has been possible to establish the scaling factors between animal hosts and humans for DNA and protein adduct formation, as well as to establish plasma and urinary biomarkers of PhIP exposure. The sensitivity of AMS is required to keep both the chemical and the radiation doses to human volunteers to levels that do not exceed commonly accepted risks. These works have led to a follow-on research to establish an AMS-based assay for quantifying PhIP-protein adducts in humans and the use of urinary PhIP-metabolites as biomarkers of PhIP exposure [28,29].

The work with heterocyclic amine carcinogen has been expanded to the development of chemopreventive strategies for reducing the amount of DNA damage following carcinogen exposure and to the quantitation of the ability of certain dietary agents to reduce the levels of DNA adducts from two heterocyclic amines, PhIP and 2-amino-3-methylimidazo [4,5-f]quinoline (IQ) [30]. It was found that chlorophyllin (a stable form of chlorophyll that is found in green leafy vegetables) and the isothiocyanate (found in broccoli) caused the greatest adduct reductions in prostate, liver and colon of rodents, presumably by modifying the metabolic processes leading to the final reactive metabolites. Turesky *et al*. performed a study to explore the use of coffee as a cancer chemopreventive agent as well [31]. They studied not only the effect of coffee on the enzymes that metabolize PhIP, but also its utility in reducing PhIP-DNA adduct levels. A 50% decrease in adduct formation was observed after 24-hour exposure in the liver of rats on the 5% coffee diet *vs*. the control group. An induction of glutathoine *S*-transferases, which are involved in the detoxication of HONH-PhIP and its reactive *N*-acetoxy intermediate might contribute to this protective effect of coffee in liver, not in extrahepatic tissues, based upon PhIP-DNA adduct formation. Various animal models were also used to study covalent binding levels of toxic compounds, including a quantitation of chromatin adducts of acrylamide in male germ cells of mice that was related to pre-implantation abnormalities in embryos [32]. The sample material available for analysis was so small that AMS quantitation could be the only reasonable route for obtaining very pure chromatin.

Very recently, AMS was successfully used to measure the kinetics and repair of DNA adducts formed by two chemotherapeutic compounds, carboplatin and oxaliplatin [33,34]. For carboplatin, AMS was used to measure carboplatin-DNA binding in purified genomic DNA and in T24 human bladder cancer cells [34]. The kinetics observed for the first time for reaction with genomic DNA revealed that the rate of carboplatin-DNA adduct formation was approximately 100-fold slower than that reported for the more potent analog cisplatin, which may explain the lower toxicity of the compound. In human bladder cancer cells exposed to carboplatin, AMS allowed a measurement sensitivity of 1 amol per 10 μg of DNA. In addition, the rate of oxaliplatin adduction to salmon sperm DNA was measured, and oxaliplatin-DNA adduct distribution was further investigated at the nucleoside level by high-performance liquid chromatography (HPLC)-AMS following enzymatic digestion [33]. Importantly, rates of cellular drug influx, efflux, DNA damage and DNA repair in cultured platinum-sensitive testicular (833K) and platinum-resistant breast and bladder (MDA-MB-231 and T24, respectively) cancer cells incubated with a subpharmacological dose of oxaliplatin (0.2 μM) were quantified and differentiated by AMS.

Radiolabeled adriamycin, also known as doxorubicin, was introduced to MCF-7 human breast cancer cells by Coldwell *et al*. [35]. Although adriamycin is an anti-cancer agent with the widest spectrum of anti-tumor activity, especially in the treatment of breast cancer and the dominant mechanism of action appears to involve impairment of topoisomerase IIα activity, the exact mechanism by which adriamycin exerts its anti-tumor activity is still uncertain. With many potential alternative mechanisms of action cited and reviewed, the technology of AMS has provided the first direct evidence of adriamycin-DNA adducts at clinically-relevant adriamycin concentrations.

Kwok *et al*. found dose-dependent binding of orthophenylphenol (OPP) fungicide to proteins in the rat urinary bladder, but found no significant covalent binding to the DNA [36]. The observed carcinogenic effect of OPP in the bladder might be due to the interference of critical cellular functions through quinone initiated oxidative stress and/or an interaction between quinones and critical sulfhydryl-containing protein targets resulting in genetic alterations or cell death. The high sensitivity of AMS helps discard the hypothesis of DNA binding, even at the low doses administered.

Two studies [37,38] looked at the genotoxicity potential of benzene by quantifying species and strain differences of the protein and DNA binding at very low doses and by testing for histone-specific binding of benzene metabolites using gel separations and MS analysis with AMS quantitations. It should be noted that no specificity toward individual histone species was found though protein adducts of benzene or its metabolites were indicated by elevated levels of ^14^C and that these studies used realistic inhalation doses. The incorporation of ^14^C was largely proportional to the density of gel staining, giving little evidence that the proteins were specific targets for selective labeling, implying high reactivity of benzene toward proteins which enables such attack to occur at multiple sites within multiple targets. In addition, Goldman *et al*. used postlabeling to eliminate the need for using a ^14^C-labeled compound directly in the biological subject in a study of benzo-pyrene adduction to DNA [39], where ^14^C-labeled acetic anhydride was used to recognize and label benzo[a]pyrene adducts on DNA.

Liu's group applied AMS to the study of the DNA adduction of several common ^14^C-labeled chemicals. One study shows that the adduction of nitrobenzene is suppressed by vitamin C, vitamin E, tea polyphenols, and other dietary substances [40]. A high-dose level of sodium benzoate (500 mg/kg of body weight) in mice resulted in higher adduction in the kidney than in the liver. The levels of DNA-benzoate adducts decayed quite rapidly initially but persisted at a low level which is relevant to chronic use of sodium benzoate [41].

The dangers of certain dietary compounds to genetic material was then expanded to include aflatoxin B_1 _(AFB_1_) adducts as measured in the colon DNA of rats and humans [42], in which the levels of AFB_1_-DNA and AFB_1_-albumin adducts were investigated by AMS, indicating that there is a linear relationship between the exposure to AFB_1 _and AFB_1_-albumin adduct formation in rats in a dose range of 0.16 ng/kg–12.3 μg/kg, and that the protein adduct levels in the rats are similar to humans.

The human metabolism of atrazine herbicide was traced in urine as a function of time for one week after a dermal exposure [43]. A highly polar metabolite, previously undiscussed, formed the largest single fraction of the excreted metabolites by the second day and continued at the same level for one week. These measurements constituted a "rescue" effort, because the ^14^C level in the urine was too low for LSC of the chromatography fractions. The fractions were easily quantified by AMS, even for a total of 1.7 fmol of ^14^C loaded on the column in the 7-day post dose sample.

Boocock *et al*. identified the human cytochrome enzyme involved in metabolizing the cancer therapeutic tamoxifen to reactive states that can lead to DNA adduction [44]. They also quantified the level of binding tamoxifen to endometrial and colon DNA in an attempt to understand the role, if any, of this drug in endometrial and colon cancer. They expanded the study to establishing if tamoxifen binds irreversibly to uterine DNA when given to women patients who were given a single therapeutic dose of ^14^C-labeled tamoxifen citrate orally ~18 hours prior to hysterectomy or breast surgery [45]. They demonstrated that after oral administration, tamoxifen forms adducts in human uterine DNA but at low numbers relative to those previously reported in women after long-term tamoxifen treatment where levels, when detected, ranged from 15,000 to 130,000 adducts/10^12 ^nucleotides.

### Neuroscience study using AMS

AMS has been used to study long-term pharmacokinetics, to identify biomolecular interactions, to determine chronic and low-dose effects or molecular targets of neurotoxic substances, to quantify transport across the blood-brain barrier (BBB) and to resolve molecular turnover rates in the human brain on the time-scale of decades.

Organophosphates, such as diisopropyl fluorophosphates (DFP), are frequently used as insecticides. DFP has been previously used as an experimental agent in neuroscience for its ability to inhibit cholinesterases and induce delayed peripheral neuropathy [46] and as an ophthalmic cholinesterase inhibitor in glaucoma treatment [47,48]. The sensitivity of AMS permitted the study of low-level (sub-toxic) exposure to acutely toxic compounds *in vivo *(DFP has an oral LD_50 _in rat of 1.3 mg/kg). Vogel's group quantitated low-dose binding of the nerve agent analog, DFP, to the plasma and brain proteins of mice as a quantifiable biomarker of multiple chemical effects from pre-exposures of parathion (PTN), permethrin (PER), and pyridostigmine bromide (PYB) [49]. They found that brain DFP binding increased by 25–40% under various pesticide pre-exposure in food, although the plasma binding concentrations did not change. A cholinergic-derived induction of NO was hypothesized to increase brain blood flow, resulting in higher delivery of DFP to the brain prior to its metabolism by copious plasma and liver estrases. Pyridostigmine produced a general 15% decrease in binding, presumably due to lower bioavailability of the food-delivered toxins arising from increased intestinal peristalsis. The effect on the permeability of the BBB to low doses of pesticide mixtures was also investigated using ^14^C-labeled DFP as a quantifiable probe of effects due to unlabeled PTN, PER and PYB separately and in conjunction [49]. The study concluded that if the increase in brain DFP level were due to increased permeability of the BBB, other toxins or pathogens might also induce increased BBB permeability with low pesticide exposure, and that the sensitivity of AMS allowed the probing of specific biochemical pathways using physiological doses, which did not perturb the natural system of the model animal.

After the emergence of Gulf War Syndrome in veterans of the 1991 Gulf War, synergistic exposures to combinations of esterase inhibitors were a hypothesized contributor, and AMS was used to examine the effect of chronic exposure to PYB (7.75 mg/kg per day in chow) on acute doses of ^14^C-labeled PER (4.75 μg/kg). At 1 h after dosing, the amount of PER in brain and spinal cord was reduced by 30% for animals receiving PYB. At 24 h, there was no difference in PER in the brain but the spinal cord had 70% less PER with PYB exposure. The levels of PER in the plasma was the same for each dose group. The sensitivity of the measurement was pg/g per equivalents in dissected tissue. Since PER and PYB are not direct competitors for enzyme binding, and the qualified effect is too large for competitive inhibition at these doses, a physiological effect such as decreased bioavailability is suggested.

**Figure 3:**
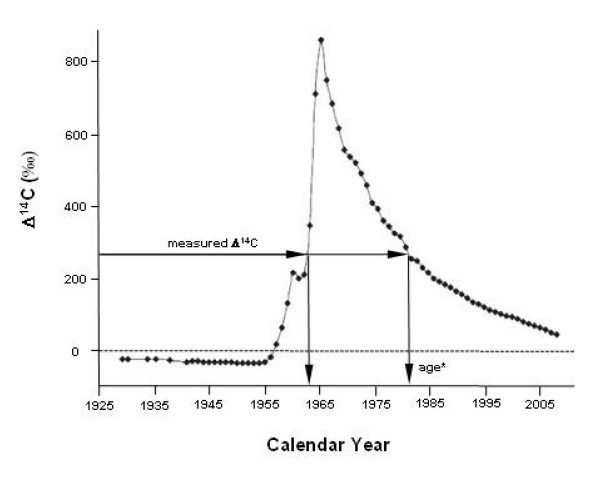
**Bomb curve used for dating recent biological materials**. The levels of ^14^C in the atmosphere have been relatively stable over long time periods, with the exception that atmospheric nuclear weapons tests in 1955–1963 added significant amounts of ^14^C to the environment. *The age of the biological material is calculated based on an assumption that the organism's biosynthesis is in isotopic equilibrium with its carbon sources. Choosing between the two dates requires additional data.

It is estimated that more than 90% of degenerate dementias are proteinopathies. *i.e*. caused by abnormal protein aggregation [50]. In Alzheimer's disease (AD), these are primarily different amyloid β (Aβ) peptides and a hyperphosphorylated form of the tau protein [51], whereas α-synuclein is implicated in Parkinson's disease, dementia with Lewi bodies and other forms of dementia [52,53]. Although numerous contributing factors have been identified, the etiology of these diseases is generally poorly understood. The bomb pulse of ^14^C (Figure 3) was used to determine the average date of formation of the major histopathological features in AD brain: extracellular senile plaques (SP), composed primarily of Aβ peptide, and intracellular neurofibrillary tangles (NFT), composed of paired helical filaments containing hyperphosphorylated tau proteins [51]. The changing ^14^C level of contemporary carbon was also used to determine the carbon 'age' of normal brain tissue (1.4 years). The SP and NFT structures have a much slower carbon turnover rate than normal tissue and are not in a formation/degradation equilibrium. The study showed that the average age of isolated SP and NFT was significantly greater than normal tissue from the same subjects (SP by 9.8 ± 4.9 years and NFT by 9.4 ± 3.8 years). Although a clear and consistent pattern of formation of NFT and SP could not be formulated from the small number of analyzed subjects, in four out of six cases, average SP and NFT or both predated the onset of symptoms by as long as 9 years. It is expected that more efficient isolation techniques that can accommodate smaller specimens from specific brain regions will produce more consistent patterns of information and such studies could provide valuable information on the etiology and progression of AD and other neurodegenerative proteinopathies.

Calcium concentration and its spatial localization and dynamics are important in many neuronal processes, such as signaling, long-term potentiation and depression [54,55], dendrite [56], and spine formation [57]. Although very sensitive fluorescent methods for quantifying [Ca^2+^] *in vivo *and in real time are well established [58,59], they require careful calibration and cannot directly distinguish between different sources of Ca^2+^. Furthermore, the Ca^2+^-sensitive dyes add a significant exogenous buffer capacity and distort the amplitude, time course and spread of [Ca^2+^] signals. Lin *et al*. in 2004 improved the sample chemistry required to extract calcium quantitatively from plasma, urine, and saliva using schemes that greatly increased the sample throughput as well as quality of the samples [60]. They studied ^41^Ca quantitation with AMS to access bone health for humans and dietary protein effects on bone resorption. It is expected to quantify changes in bone resorption using ^41^Ca arising from ^3^H- or ^14^C-labeled pharmaceuticals. The value of ^41^Ca quantitation for cancer, aging, and nutritive research is becoming better recognized, and AMS technique will maintain its present dominance in high throughput measurement of this important biomedical tracer isotope.

### Pharmacokinetic study using AMS

The benefits of using AMS for the analysis of samples derived from radiotracer studies include pharmacokinetic studies with humans. The maximum radioactive dose that can be administered to humans depends on the residence time in the body and whether it is accumulated in specific tissues. Under certain circumstances, *i.e*. when there is a long pharmacokinetic half-life, the amount of radioactivity that can be administered is below the capabilities of LSC. Since the advent of biomedical AMS, it has been possible to conduct radiotracer studies in humans, with the administration of such low levels of ^14^C that they are, from a regulatory point of view, considered non-radioactive.

Furthermore, in the development of a new drug, there may be a number of candidate compounds available to go forward into clinical trials. The classical selection process involves conducting a series of modeling experiments using *in vitro*, cell-based or *in silico *techniques and some experiments with small numbers of laboratory animals. Through a process known as allometric scaling, a prediction is made of the pharmacokinetics of the candidate drug in humans. However, allometric scaling has occasionally failed to adequately predict the behavior of the drug in humans. In an ideal case, all the candidate drugs would be dosed to humans and selection made on the basis of true *in vivo *data. Practically, however, such an approach would be prohibitively expensive due to the enormous amount of preclinical toxicology safety testing required.

An alternative approach, therefore, has been suggested [10] where very small amounts of drugs, in the microgram range, are administered. With such trace doses, the toxicological safety tests required are vastly reduced [22]. Hence, it is possible to dose a range of candidate drugs to humans and select the one with optimum pharmacokinetics to take forward for further clinical evaluation. The major barrier to this approach is the extremely sensitive methods of detection are required in order to quantitate the drug and its metabolites in plasma and excreta following the administration of such a small microdose.

AMS, an innovation technology for this purpose, has made it possible to administer such low amounts of ^14^C-labeled candidate drugs to humans and still retain sufficient levels of analytical sensitivity to determine its metabolism and pharmacokinetics, even though the amount of radioactivity that can be administered to humans is limited owing to the radiation exposure, not surprisingly. This technique is, however, very much in its infancy and it is not known, for the majority of drugs, whether the pharmacokinetics will be sufficiently linear so that the pharmacokinetics observed at the microdose will be predictive of those at the therapeutic dose. It is important, therefore, to study comparative pharmacokinetics of a drug candidate at high and low doses to establish the validity of the "microdosing" concept, that is, a human microdosing study comprises the administration of a sub-pharmacological/sub-therapeutic dose of novel drug candidate(s) in order to gain essential pharmacodynamic and pharmacokinetic information [10,11]. To date, a major trial is underway financed by a group of pharmaceutical companies to test this theory.

In 2004, Sandhu *et al*. reported [61] the first description of the full pharmacokinetic profile of a drug candidate assessed and of the comparisons of the kinetics of a pharmaceutical compound at pharmacological versus sub-pharmacological doses employing microdosing strategies, in order to address the unresolved issue of whether the pharmacokinetics determined following a microdose are representative of those following a conventional (pharmacological) dose. They successfully validated and utilized the technique of AMS to study the pharmacokinetics and disposition in dogs of a preclinical drug candidate after oral and intravenous administration. They emphasized in the paper that only the exceptional sensitivity of AMS can provide a pharmacokinetic profile of the drug candidate, even following a microdose, which reveals aspects of the disposition of the agent that are inaccessible by conventional techniques. Li *et al*., on the other hand, designed a new drug delivery microelectromechanical systems (MEMS) to deliver tracer molecules as well as a therapeutic agent *in vivo *and evaluated their spatial and temporal release profiles using AMS [62].

### Nutritional study using AMS

It is becoming apparent that all humans do not identically respond to either diets or medicines, so their needs differ according to differences in their genetic information and physiological status. AMS may be particularly useful in obtaining accurate spatial information and low-level detection of essential and nonessential bioactive food components (nutrients) and their metabolites, and in enhancing the understanding of the impact of nutrient/metabolite and biomolecular interactions. The fate and distribution of vitamins at physiological concentrations within healthy humans of all ages, for instance, had not been quantified prior to the use of AMS [63]. Vitamin research was performed including mathematical statistics and modeling using the high density human kinetic data to explore the parameter space of kinetic modeling [64,65].

The initial study involved sub-physiological doses of folic acid, which is especially important to the health of young mothers, but which had been studied only in elderly ill human subjects. AMS tracing doses contain a few hundred nanoCurie of ^14^C or less, even for highly recirculated nutrient chemicals, exposing the volunteer subject to less radiation damage than is obtained within 10 minutes in a commercial air flight. This is a commonly accepted level of radiation exposure, even among pregnant women, to whom a better understanding of their true folate needs is important. An isotopic form of folate was required to distinguish the dosed material from the greater amount of endogenous folate, but stable isotopic approaches have not been able to follow single physiologic doses for more than a few days in human volunteers [66]. Other studies used chronic isotopic dosing to obtain turnover and kinetic elimination measures, but do not provide detailed kinetic profiles [67]. The initial data quickly showed that folate was an effective label for the study of red blood cell production, lifetime and elimination [43], which has been approached by hematologists about the possibility of using this pulse-chase labeling mode to study the red blood cell lifetimes in disease states such as sickle-cell anemia or malaria. Lin *et al*. summarized the 6-months pharmacokinetic data from 13 human subjects with a median age of 24, including 7 women and suggested a connection between liver disease and folate deficiency, as revealed by detailed compartmental modeling of the entire high-density data sets from all 13 subjects [60,68]. These works may explain the world's highest incidence of neural tube birth defects along the southern Rio Grande [69] as arising from the endemic levels of hepatitis [70], rather than the apparently unrelated low folate intake [71], or common polymorphisms within the Hispanic population [72]. Relations between cirrhosis and homocystenemia, a risk factor in heart disease, also derived from the large recycling of demethylated folate through bile and back into the liver for remethylation [73,74].

The small samples used in AMS can provide high data density. AMS has the high sensitivity for long-term kinetic analysis to give detailed elimination information. Thus, only AMS could result in a model sufficiently detailed to reveal the hidden variables possibly responsible for these health effects. Dueker *et al*. showed that naturally produced β-carotene could be used at low doses to judge the vitamin A potential of carotene [75]. Reverse-phase HPLC was used with AMS to quantify plasma metabolites of the carotene in 99 hours after dosing. An unidentified acidic metabolite, possibly an epoxide, was found to comprise 15% of the circulating acidic fraction, and the metabolism of β-carotene to retinol and retinyl esters was surprisingly enhanced by a vitamin A supplementation that should decrease the need for carotene-derived retinal [76,77]. An increase in carotene absorption at the expense of intestinally produced retinyl esters was also found.

### Other important research using AMS

Non-covalent equilibrium binding was quantified by AMS in two studies seeking to develop more sensitive immunoassays (IA) [78,79]. Lu *et al*. developed an AMS-IA to one species of the parathyroid hormone related protein that is a biomarker of prostate cancer [78], and Shan *et al*. showed that AMS increased sensitivity and quantitation over already sensitive ELISA-IA's for atrazine and dioxin without resulting in a waste stream that exceeded the government's definitions of radioactive materials [79]. These studies set the stage for further development of non-covalent labeling strategies that are not as straightforward as the previously listed covalent binding work, and may serve as models for the development of sensitive and "nonradioactive" IA for peptides, including polypeptide tumor markers.

The two IA efforts above are also forms of postlabeling recognition of specific protein moieties and chemicals in biological solutions. Miyashita *et al*., on the other hand, demonstrated highly sensitive protein sequencing by Edman degradation, which is an impetus for developing methods of analyzing extremely small amounts of biological systems [80]. Their method is expected to be applicable to the sequencing of proteins from cell culture and illustrates a path to more general methods for determining *N*-terminal sequences with high sensitivity.

**Figure 4:**
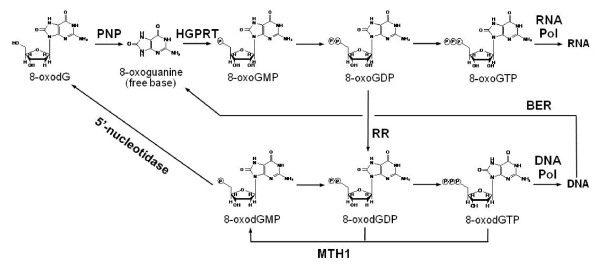
**Proposed mechanism of 8-oxo-7,8-dihydro-2'-deoxyguanosine (8-oxodG, a well known DNA oxidative damage biomarker) metabolism in MCF-7 human breast cancer cells, where PNP stands for purine nucleoside phosphorylase, HGPRT for hypoxanthine-guanine phosphoribosyltransferase, RNA Pol for RNA polymerase, DNA Pol for DNA polymerase, RR for ribonucleotide reductase, and BER for base excision repair, respectively**. MTH1 is a pyrophosphatase capable of cleaving the pyrophosphate either from 8-oxodGTP or 8-oxoGTP, thus preventing the accumulation of a potentially mutagenic species.

Biomedical applications of AMS were expanded to the study of DNA damage/repair. An AMS method was developed for rates of *in vivo *incorporation and repair of an 8-oxo-7,8-dihydro-2'-deoxyguanosine (8-oxodG), a well known DNA oxidative damage biomarker [81-83]. These studies were performed to determine if 8-oxodG can be phosphorylated and incorporated into DNA from oxidation of the nucleotide pool. The composition of the radiolabeled nucleotides incorporated into DNA from the ^14^C-labeled 8-oxodG dosed cells was determined by nucleoside digestion followed by HPLC. Digestion of the DNA to nucleosides followed by separation with HPLC allowed determination of the composition of the radiolabeled nucleosides in the purified DNA. Each chromatogram shows a single peak that coelutes with an authentic standard of 8-oxodG. The single peak observed is also confirmation of the quality of the DNA digest and purification conditions, which were optimized to avoid artifactual 8-oxodG oxidation. The data collectively indicate that in the cells studied the nucleotide pool can be a significant source of 8-oxodG for incorporation into genomic DNA. Importantly, it was found that the rate of incorporation of 8-oxodG is approximately equal to that of dG and that the maximum concentration achieved was ~2 per 10^7 ^normal nucleotides, a level approaching that of background 8-oxodG levels in most cell types. Interestingly, it was unexpectedly found that radiocarbon from ^14^C-labeled 8-oxodG was also incorporated into RNA, which was followed by a mechanistic analysis of several pathways by which 8-oxodG is converted to nucleotide triphosphates and incorporated into both DNA and RNA [81], allowing to propose an 8-oxodG metabolic mechanism in MCF-7 human breast cancer cells, as illustrated in Figure 4. A new approach is now opened to the mechanistic study of measuring the kinetics of small molecule fates at a very low level of detection with high precision and observing the initiating events in the nucleobase modification that lead to carcinogenesis or other diseases.

AMS was also used as a core technique to verify that 5'-methylthioinosine is an active nucleic acid precursor in *Plasmodium falciparum *which is unable to synthesize purine bases and relies on purine salvage and purine recycling to meet its purine needs [84]. To understand the purine pathways of malaria, they characterized the activities of adenosine deaminase and purine nucleoside phosphorylase from *P. falciparum *which have catalytic specificities that allow them to use methylthiopurines and therefore to function in both purine salvage and methylthiopurine recycling. By using AMS, they showed this pathway is active in *P. falciparum *cultured in human erythrocytes.

Retrospective birth dating of cells in humans was achieved based on the AMS measurement, which is a generally applicable strategy that can be used to measure cell turnover in man under physiological and pathological conditions [85]. They took advantage of the fact that testing of nuclear weapons resulted in a dramatic global increase in the levels of the isotope ^14^C in the atmosphere, followed by an exponential decrease after 1963, as shown in Figure 3[86-88], and they showed that the level of ^14^C in genomic DNA closely parallels atmospheric levels and can be used to establish the time point when the DNA was synthesized and cells were born. They also found that the strategy can be used to determine the age of cells in the cortex of the adult human brain leading to the conclusion that whereas nonneuronal cells are exchanged, occipital neurons are as old as the individual, supporting the view that postnatal neurogenesis does not take place in this region.

In addition to the novel forensic application of AMS above, the age at death of individuals, an important step in their identification, was also established with high precision by AMS analysis of dentition [89]. It was demonstrated that the amount of radiocarbon present in tooth enamel as a result of nuclear bomb testing is a remarkably accurate indicator of when a person was born, since the enamel of individual teeth contains 0.4% carbon and there is no turnover of enamel after it has been laid down, *i.e*. the ^14^C concentration reflects that in the atmosphere at the time of enamel formation. They, therefore, measured the ^14^C content of tooth enamel and related it to the known concentrations in the atmosphere in different years to establish the year of tooth formation. The date was then related to the known age for enamel deposition of individual teeth to establish the person's year of birth, resulting in a remarkably precise estimate of age for 22 individuals (R^2 ^= 0.99). The average systematic deviation from the correct value was +0.2 years, and the average absolute error for individual measurements was 1.6 ± 1.3 years, indicating that the precision is substantially higher than that obtained by other available methods.

The technology of AMS has been utilized to establish the dynamics within the stable population of adipocytes in adults, by measuring adipocyte turnover and analyzing the integration of ^14^C derived from nuclear bomb tests in genomic DNA [85,90]. Spalding *et al*. demonstrated using AMS that approximately 10% of fat cells are renewed annually at all adult ages and levels of body mass index, although the number of adipocyte is set during childhood and adolescence. Their results suggest that neither adipocyte death nor generation rate is altered in early onset obesity, and that a tight regulation of fat cell number in this condition during adulthood.

For practical purposes, rapid methods have been developed to convert organic species into forms compatible with direct introduction to the spectrometer for ^14^C analysis to perform the AMS measurements more efficiently [12]. Ognibene *et al*. developed a high throughput modification of the reduction stage for carbon sample preparations. The technique uses custom-made septa-sealed reaction vessels for the trapping, purification, and reduction of combustion gases to the desired elemental carbon on an iron-group catalyst. The combustion gases can come from sealed combustion tubes that are the most efficient process for large numbers of mg-sized samples.

### Conclusions and future prospects

While the initial themes of biomedical research with AMS involved primarily the kinetics and binding of carcinogenic toxins and focused on toxicokinetics and toxin metabolism with new initiatives in nutrition and immunoassays, scientists have now expanded the study of kinetics and dynamics directly in humans for disease [91,92], nutritional [75], and pharmaceutical [16] research, since AMS is now a proven sensitive and robust method for quantifying rare isotopes in biological systems. Moreover, AMS has been utilized for the detection of biomarkers or molecular targets of relevance to nutrition and cancer and other chronic diseases. This opens up the whole area of biomarker studies where currently only changes to the size of a metabolic pool are measured. For example, a decrease in a metabolic biomarker might be due to increased catabolism or decreased anabolism. Using a trace dose of ^14^C-labeled precursor, the turnover rates of the biomarker could be determined without unduly exposing the volunteers to adverse levels of radioactivity. In theory, any and all endogenous components of a biological system can be quantified by versatile AMS coupled with amol radiolabeled-isotope detection capability if that system is uniformly labeled by an isotope. Under this condition, all structural, signaling, and nourishing components become quantifiable at amol levels by AMS. Such an approach depends on quantitative isolation of the chosen components from other isotopically labeled materials. Cultures can be commonly grown on isotopically enriched substrates, usually to produce specific proteins or lipids for further tracing experiments. More frequently, specifically labeled precursors can be added to cultures to enhance the isotopic signal of chosen components. These isotopic enrichments are akin to tracing specific compounds that cannot reveal the entire biochemical balance of a system. Biomolecules that are stable over those 20 to 50 years can also be quantified as being retained from a uniformly labeled system. Any biochemical pathway can be virtually quantified by AMS if a sufficiently specific labeling procedure can be found. It is also expected quantitative postlabeling strategies can be developed for oxidative and other molecular modifications or functions as examples of AMS application to a very broad field of biomedical research. The unique analytical methods are expected to provide the scientific proof-of-principle framework that will proceed through increasing levels of complexity, to broaden the biomedical applications of AMS to problems in biochemistry, cell biology, developmental biology, pharmacology, immunology and others. The knowledge obtained is also expected to be combined with other biological studies to achieve more complete pictures of several important biological processes.

Although use of these techniques was not widespread because of the high instrumentation costs of commercially available systems and the need for qualified physicists to operate the instrument (at the beginning of the new millennium there were, world-wide, approximately 50 labs engaged in AMS research), the situation is now far improving. At present, there are several companies for the commercial exploitation/analysis of AMS and it is also expected that the instrumentation costs will be less than $ 2 M.

AMS has been called an enabling technology, and especially AMS for ^14^C analysis has become more accessible and inexpensive, making the biomedical application of AMS no more difficult than other tracing and quantifying methods now used in routine biomedical research. There are likely to be many more applications to biomedical science for this technology in the future, so far never-thought-of.

## Competing interests

The author declares that he has no competing interests.
