# Recent advances in biomedical applications of accelerator mass spectrometry

**DOI:** 10.1186/1423-0127-16-111

**Published:** 2009-12-09

**Authors:** Sang Soo Hah, Paul T Henderson, Kenneth W Turteltaub

**Affiliations:** 1 Department of Chemistry and Research Institute for Basic Sciences, Kyung Hee University, Seoul 130-701, Korea; 2 Physics and Life Sciences Directorate, Lawrence Livermore National Laboratory, Livermore, CA 94551, USA

## Abstract

After publication of our article, it was noted that we inadvertently failed to include the complete list of authors. The full list, including co-authors, has now been added and the Authors' contributions and Competing interests sections modified accordingly.

## Correction

After publication of our article [1], it was noted that we inadvertently failed to include the complete list of authors. The full list, including co-authors, has now been added and the Authors' contributions and Competing interests sections modified accordingly.

## Competing interests

The authors declare that they have no competing interests.

## Authors' contributions

SSH wrote the manuscript. PTH and KWT are long-time collaborators, who supported the initiation of this review. All authors read and approved the final manuscript.

## References

[B1] Hah SS Recent advances in biomedical applications of accelerator mass spectrometry J Biomed Sci 2009 16 54 10.1186/1423-0127-16-54 19534792 PMC2712465

